# Comprehensive landscape of the ST3GAL family reveals the significance of ST3GAL6-AS1/ST3GAL6 axis on EGFR signaling in lung adenocarcinoma cell invasion

**DOI:** 10.3389/fcell.2022.931132

**Published:** 2022-08-26

**Authors:** Jiaxuan Li, Yiming Long, Jingya Sun, Jiajun Wu, Xiao He, Simei Wang, Xiongbiao Wang, Xiayi Miao, Ruimin Huang, Jun Yan

**Affiliations:** ^1^ Department of Respiratory Medicine, Putuo Hospital, Shanghai University of Traditional Chinese Medicine, Shanghai, China; ^2^ Shanghai Institute of Materia Medica, Chinese Academy of Sciences, Shanghai, China; ^3^ University of Chinese Academy of Sciences, Beijing, China; ^4^ Department of Laboratory Animal Science, Fudan University, Shanghai, China; ^5^ School of Chinese Materia Medica, Nanjing University of Chinese Medicine, Nanjing, China; ^6^ Interventional Cancer Institute of Chinese Integrative Medicine, Shanghai University of Traditional Chinese Medicine, Shanghai, China

**Keywords:** lung adenocarcinoma, sialyltransferase, prognostic analysis, ST3GAL6, ST3GAL6-AS1

## Abstract

Sialylation aberration has been implicated in lung cancer development by altering signaling pathways. Hence, it is urgent to identify key sialyltransferases in the development of lung adenocarcinoma (LUAD), which is a common malignant subtype of non-small cell lung cancer. Herein, by systematically investigating the expression levels of ST3GAL family members in several public databases, we consistently found the frequent downregulation of ST3GAL6 in LUAD samples. Its downregulation is significantly negatively associated with stage, and significantly reduced in proximal-proliferative molecular subtype and predicts poor clinical outcomes. By protein–protein interaction network analysis and validation, we found that ST3GAL6 deficiency promotes LUAD cell invasiveness with the activated EGFR/MAPK signaling, accompanied by the elevated expression levels of matrix metalloproteinases 2 and 9, which can be partially reversed by EGFR inhibitor, gefitinib. Additionally, the ST3GAL6 level was positively regulated by ST3GAL6-AS1, an antisense long non-coding RNA to its host gene. The downregulation of ST3GAL6-AS1 also heralds a worse prognosis in LUAD patients and promotes LUAD cell invasiveness, recapitulating the function of its host gene, ST3GAL6. Altogether, ST3GAL6-AS1-regulated ST3GAL6 is a frequently downregulated sialyltransferase in LUAD patients and negatively regulates EGFR signaling, which can serve as a promising independent prognostic marker in LUAD patients.

## Introduction

Lung cancer remains the most common cancer in the world, with 228,820 cases and 135,720 deaths reported last year according to the International Agency for Research on Cancer (IARC) statistics on new cancer cases and death rates in 2020 (Siegel et al., 2020). The lung adenocarcinoma (LUAD) subtype of non-small cell lung cancer predominates in these cases, and the majority of them are non-smokers (Rivera and Wakelee, 2016; Herbst et al., 2018). This subtype is also associated with highly frequent mutations of EGFR (Calvayrac et al., 2017). Unfortunately, the clinical use of EGFR inhibitors does not significantly improve survival in LUAD patients, suggesting that there may exist other regulations to affect EGFR activity, such as post-translational modifications. Glycosylation modification is one of such post-translational modifications and has been implicated in the development of various cancer types by affecting protein conformation and protein–protein interaction (Lemjabbar-Alaoui et al., 2015; Takahashi et al., 2016; Yang et al., 2021b). However, the key glycosylation-associated molecule events in the LUAD development have not been fully elucidated.

As one of the important glycosylations, sialic acid modification is a terminal sugar modification, in which negatively charged sialic acids consisting of nine-carbon monosaccharides are linked by means of either α2,3-, α2,6- or α2,8-primarily by sialyltransferase (Pearce and Läubli, 2016; Yang et al., 2021a). In mammalian cells, the ST3GAL family, which consists of six family members, is responsible for the specific catalysis of the transfer of sialic acid in the α2,3- junction (Harduin-Lepers et al., 1995; Harduin-Lepers et al., 2001). A highly abnormal sialylation on the glycosyl-terminal surface of tumor cells facilitates signal transduction and cell-to-cell interaction (Hudak et al., 2014; Wang et al., 2020). For instance, an abnormal sialylation of key signaling pathway components, such as TNFR1 and TGF-β1, or Lewis X antibody and Lewis A antigen, can promote tumor progression and metastasis (Gretschel et al., 2003; Pérez-Garay et al., 2010; Yeo et al., 2019). By the unsupervised hierarchy clustering analysis on glycogenes, our previous study has identified ST3GAL6 as a poor prognostic biomarker in bladder cancer (Dalangood et al., 2020), suggesting that we can identify key drivers from glycogenes for tumor aggressiveness. However, the key sialyltransferase for the regulation of sialylation during LUAD development remains largely unknown.

Herein, we first comprehensively analyzed the expression levels of six ST3GAL family members with clinicopathological parameters, molecular subtypes, prognoses, and status in LUAD patients in several public datasets. Furthermore, we chose ST3GAL6, which showed a consistent downregulation in LUAD samples, to analyze its associated gene clusters by GO, KEGG, and protein–protein interaction (PPI) network analyses. In the end, we investigated its biological role in the activation of EGFR signaling and identified its upstream regulator in LUAD cells.

## Methods and materials

### Oncomine

The expression difference of ST3GAL family members in LUAD was analyzed by the Oncomine website (www.oncomine.org). We selected expression differences in lung cancer and the further selected LUAD database results were presented. The data parameters: *p* value: 0.05, fold change: 1.5, gene rank: All, data type: mRNA, analysis type: cancer vs. normal tissue. Student’s t-test was used for gene expression differences.

### Dataset selection

The LUAD gene sets and clinical profiles were downloaded from the TCGA Genomic Data Commons Data Portal (https://portal.gdc.cancer.gov/), cBioPortal (http://www.cbioportal.org/), and NCBI GEO databases (https://www.ncbi.nlm.nih.gov/geo/): GSE31210 (Yamauchi et al., 2012), GSE68465 (Shedden et al., 2008), and GSE72094 (Schabath et al., 2016).

### Gene expression and molecular subtype analysis in lung adenocarcinoma

In order to assign molecular subtypes of the LUAD patients, i.e., TRU (terminal respiratory unit), PP (proximal-proliferative), and PI (proximal inflammatory) as previously reported, a nearest centroid subtype predictor including 506 genes together with Pearson correlation were utilized (Cancer Genome Atlas Research Network, 2014). The expression index of the ST3GAL members were subsequently used to draw boxplots, and the statistical significance of differences in the multiple groups was determined using one-way ANOVA test followed by Tukey’s multiple comparisons test.

### Survival analysis

The overall survival (OS), recurrence-free survival (RFS), and disease-specific survival (DSS) of the ST3GALs were analyzed by using the Kaplan–Meier survival analysis with log-rank test. The clinical samples were categorized into a high- or low-expression group for the prognostic survival analysis, according to the FPKM value of each gene, showing the prognosis correlation of the expression levels of the ST3GALs, log-rank *p* < 0.05 was regarded as a statistically significant difference. Univariate and multivariate Cox regression models were conducted to investigate whether the ST3GALs can be regarded as an independent factor to predict patient prognosis by using the “survival” and “survminer” packages in R, respectively. The hazard ratio (HR) and 95% confidence interval (CI) were calculated to define the significance of gene relationships with OS/DSS/RFS.

### Gene ontology and kyoto encyclopedia of genes and genomes pathway analysis

We looked for genes co-expressed with ST3GAL6 on the cBioPortal website. Finally, a total of 1,141 genes (858 positively correlated genes and 283 negatively correlated genes) with Spearman’s Correlation > 0.3 and *p* < 0.05 were selected as genes with significant correlation with the ST3GAL6 expression for analysis. The Gene Ontology (GO) enrichment analysis and Kyoto Encyclopedia of Genes and Genomes (KEGG) pathway analysis were performed to explore the functional annotation and signaling pathways of the selected ST3GAL6-correlated genes by the R package’s “cluster profiler” (Yu et al., 2012) and “Path view” (Luo and Brouwer, 2013), respectively, adjusted *p* < 0.05 was regarded as statistically significant. When processing the results, we sorted the significant GO terms of the biological process (BP), molecular function (MF), and cellular component (CC) by adjusting the *p* value from small to large separately, and select the top ten for display. All of the significant KEGG pathways were shown by a bubble diagram. Finally, the bioinformatics online tool (http://www.bioinformatics.com.cn) was applied to visualize these results.

### Genomic mutation analysis

The cBioPortal online tool was used to investigate mutation frequency and mutation type of the ST3GAL members in LUAD.

### The human protein atlas database

Immunohistochemical (IHC) results of pathological sections from normal and LUAD patients were obtained from an online database: The Human Protein Atlas (https://www.proteinatlas.org).

### Protein–protein interactions network analysis

The PPI network analysis in this article came from GeneMANIA (http://www.genemania.org) and STRING (https://string-db.org/). We selected LUAD patients from these two databases for PPI analysis of the ST3GAL6 gene.

### Tissue microarray and IHC analysis

The tissue microarray (Cat. #: HLugA180Su04) was purchased from Outdo BioTech (Shanghai, China), accompanied with the clinicopathological information. The tissue microarray contains 88 paired LUAD and adjacent normal lung specimens, with 4 unpaired LUAD specimens. IHC staining was performed as described previously (Dalangood et al., 2020). The ST3GAL6 antibody was listed in Supplementary Table S1. The IHC scores were independently evaluated by two individuals. The final score of the ST3GAL6 staining was the multiplication of the positive ratio value (0–3) and the intensity value (0–3) of the ST3GAL6 immunoreactive cells.

### Cell culture and RNA interference

The LUAD cell lines A549 and HCC827 were derived from the Cell Bank of Type Culture Collection, Chinese Academy of Sciences (Shanghai, China). The cells were cultured in an RPMI 1640 medium (Invitrogen, Carlsbad, CA, United States) at 37°C at 5% CO_2_. The medium contained 10% FBS and penicillin/streptomycin (Invitrogen). An siRNA interference assay was performed with siRNAs targeting ST3GAL6-AS1 and a negative control (siNC). These siRNAs were transfected into the A549 and HCC827 cells by the Lipofectamine RNAi MAX Reagent (Invitrogen). The shRNA sequence-targeting ST3GAL6 and the construction of the lentivirus were designed and manufactured by Genechem Co., Ltd. (Shanghai, China) The screening process for virus infection and cell resistance was carried out according to the manufacturer’s instruction. The siRNA sequences and shRNA target sequences were listed in Supplementary Table S1.

### RNA extraction and quantitative reverse transcription PCR

Total RNA was extracted by Trizol (Invitrogen) and 1 μg total RNA was reversely transcribed into cDNA through the reverse transcription kit (R233-01; Nanjing Vazyme Biotech Co., Ltd., Nanjing, Jiangsu, China). RT-PCR analyses were performed by ChamQ SYBR qPCR Master Mix (Q341-02; Vazyme) on a Light Cycler 96 detection system (BioRad, Berkeley, CA, United States). mRNA levels were analyzed and calculated by using the ΔΔCt method and normalized with GAPDH mRNA levels. Each experiment was repeated three times independently. The primers for qRT-PCR were listed in Supplementary Table S1.

### Western blotting

Total cell protein was extracted using a RIPA lysis buffer and centrifuged at 4°C. Protein concentration was determined by a BCA kit (Tiangen, Beijing, China) and separated using 10% SDS-PAGE electrophoresis. The separated proteins were transferred to 0.45 μm nitrocellulose membranes (Millipore, Billerica, MA, United States). The membranes were blocked with 5% BSA and then incubated overnight at 4°C with primary antibodies, which were listed in Supplementary Table S1. The HRP-conjugated anti-rabbit or anti-mouse antibodies (1/5,000, Cell Signaling Technology, Beverly, MA, United States) was incubated at room temperature, after being rinsed with the PBST solution three times. The membranes were visualized by a high-sig ECL Western blotting substrate (Tanon, Shanghai, China) and detected by an automatic chemiluminescence image analysis system (Tanon, Shanghai, China).

### Transwell assay

The invasion assay was performed using a Corning Growth Factor-Reduced Matrigel (#354230; Corning, NY, United States) and 24-well Transwell chambers (8 μm; JET BIOFIL, Guangzhou, Guangdong, China). 1 mg/ml of Matrigel diluted in a 100 μl serum-free RPMI-1640 medium was coated on the upper chambers first. After 4 h of incubation of the wells at 37°C to form continuous thin layers of Matrigel, 5×10^4^ tumor cells were re-suspended in the 100 μl serum-free RPMI-1640 medium and seeded into the upper chambers. 600 μl complete RPMI-1640 containing 10% FBS was added to the bottom chambers. After 12–16 h of incubation, the top chambers were taken out, the invading cells were fixed in 4% paraformaldehyde, stained with 0.1% crystal violet for 15 min. For the Gefitinib treatment, A549 or HCC827 cells were pretreated with 5 or 1 µM Gefitinib for 24 or 16 h, followed by a 16-h Transwell assay with continued Gefitinib treatment. For the SB-3CT treatment experiment, A549 cells were pretreated with 10 μM SB-3CT for 24 h, followed by a 16-h Transwell assay with continued SB-3CT treatment. Photos were taken under a Leica DM6B Fluorescent Microscope (DM6B#; Leica Microsystems GmbH, Wetzlar, Hesse, Germany).

### Cell viability assay

A cell viability assay was performed as previously described (Shen et al., 2017; Dalangood et al., 2020). The A549 and HCC827 knockdown group and control group cells were seeded into 96-well plates at 3.0 × 10^3^ cells or 4.5 × 10^3^ cells/well and cultured for 24 h. Then, the cells were treated with the indicated concentrations of Gefitinib for 3 days. Cell survival was assessed by the SRB (Sulforhodamine B) or MTT [3-(4,5-dimethylthiazol-2-yl)-2,5-diphenyltetrazoliumbromide]. Synergy H1 hybrid (BioTek, United States) was used to measure the absorbance at 560 nm (A560; SRB assay), and the absorbance at 490 nm (A490) and A680 were used as the reference (MTT assay). The half-maximal inhibitory concentration (IC50) was calculated by using the GraphPad Prism 7.0 software (GraphPad Software Inc., La Jolla, CA).

### Statistical analysis

Welch’s *t* test and Student’s *t* test were used among clusters and variables. Pearson correlation was used to determine the correlation between gene expressions. The OS, RFS, and DSS were estimated by Kaplan–Meier (log-rank test) method. Bioinformatics-related statistical methods have been described in the previous sections. Statistical analysis was performed using the GraphPad Prism 7.0 software (GraphPad Software Inc.). Data were presented as mean standard deviation (SD) from at least three independent experiments. In all analyses, *p* < 0.05 was considered statistically significant.

## Result

### Expression levels of the ST3GAL members in lung adenocarcinoma compared with normal lung samples

**FIGURE 1:**
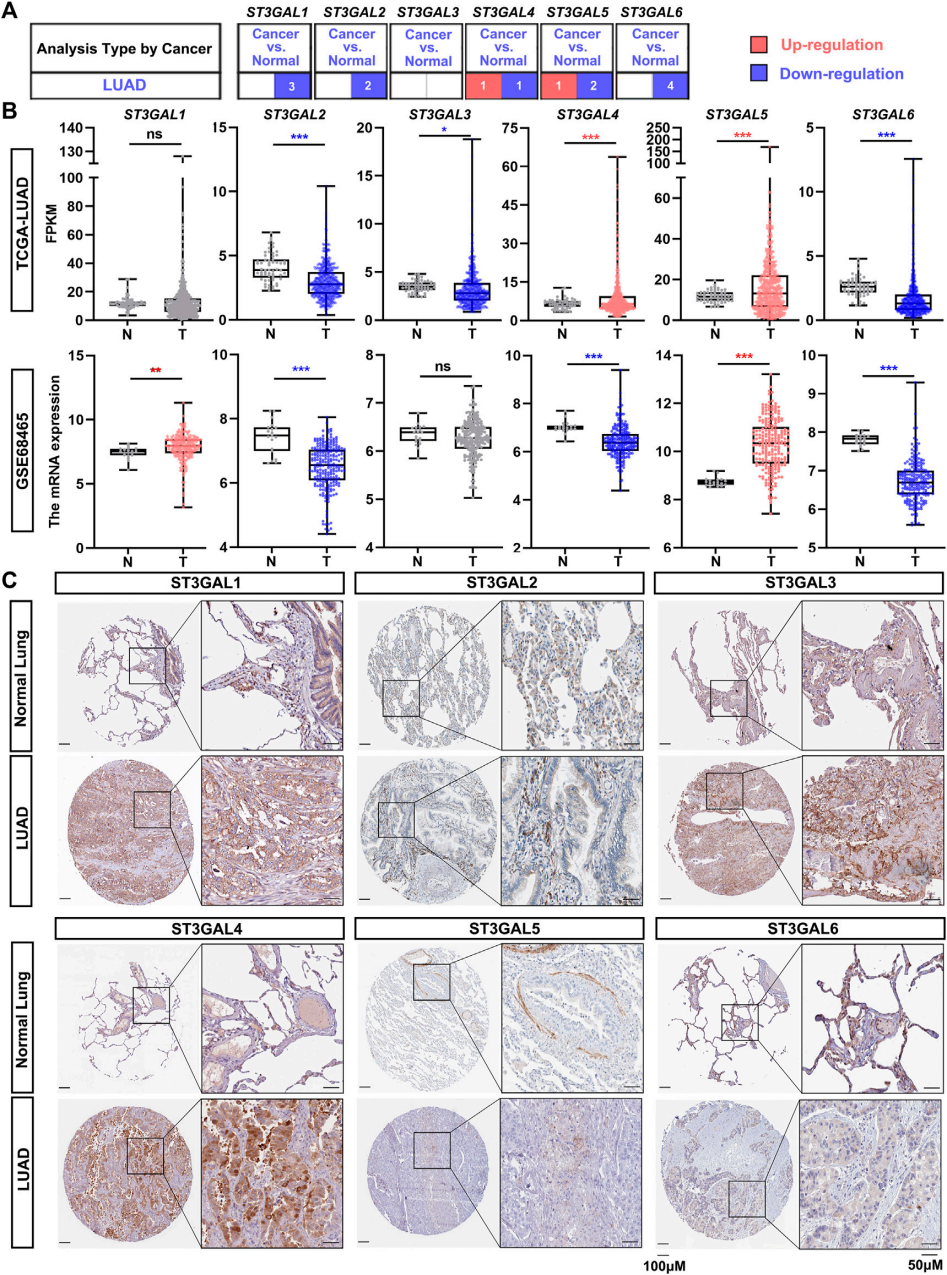
The expression levels of the ST3GAL members in LUAD samples. **(A)** The expression levels of the ST3GAL family members in normal lung samples and LUAD samples from the Oncomine database. The upregulation in LUAD samples is shown in red, while the downregulation is shown in blue. The digits in the red or blue block indicate the number of the datasets. The data parameters: *p* < 0.05, fold change: 1.5, gene rank: All, data type: mRNA, analysis type: cancer vs. normal tissue. **(B)** Comparison of the expression levels of ST3GAL family members in tumor (T) and normal (N) in TCGA-LUAD and GSE68465 datasets. T, Tumor tissues; N, Normal tissues; TCGA-Normal (*n* = 59), TCGA-Tumor (*n* = 535); GSE68465-Normal (*n* = 19), GSE68465-Tumor (*n* = 443). Dots in red indicate that the gene is upregulated; while dots in blue mean that the gene is downregulated in the LUAD samples. Welch’s *t* test, ∗, *p* < 0.05; ∗∗, *p* < 0.01; ∗∗∗, *p* < 0.001; ns: no significance. **(C)** IHC results of six ST3GAL family members in histological sections of normal lung and LUAD tissues. The representative IHC staining data were obtained from Human Protein Atlas. Scale bar, 50 μm (on the right) and 100 μm (on the left).

To explore the expression changes and potential prognostic value of the ST3GAL family members in LUAD patients, we first compared the expression levels between normal and cancer tissues in the Oncomine database. The results showed that ST3GAL1, 2, and 6 were consistently and significantly downregulated in tumor tissues, compared with normal samples (Figure 1A; Table 1). Next, we chose the TCGA-LUAD and GSE68465 datasets with a larger cohort of LUAD patients for further confirmation. The expression levels of ST3GAL2 and 6 were significantly decreased in LUAD samples, while the expression of ST3GAL5 was higher in LUAD samples than in normal tissues in both of these two independent datasets (Figure 1B). To investigate whether the expression change is due to their expressions in epithelial cells, we searched the IHC data from the Human Protein Atlas database. As shown in Figure 1C, the representative IHC data demonstrated that ST3GAL2 and 6 were expressed in normal lung epithelial cells, but strikingly downregulated in LUAD cells, whereas ST3GAL4 and ST3GAL5 were weakly expressed or barely detected in normal lung epithelial cells, but increased in the LUAD cells.

**TABLE 1:** The significant changes of the expression levels of the ST3GAL family members between LUAD and normal lung tissues (Oncomine).

Gene symbol	Type of LUAD vs. normal lung tissue	Fold change	*p* value	*t* test	Normal (n)	LUAD (n)	Source and/or references
***ST3GAL1***	LUAD	−3.441	8.32E-4	−3.625	17	132	Bhattacharjee lung statistics Aloia et al. (2015)
	LUAD	−1.998	0.002	−3.131	10	86	Beer lung statistics Guerrero et al. (2020)
	LUAD	−1.519	9.58E-6	−4.675	65	45	Hou lung statistics Liang et al. (2013)
***ST3GAL2***	LUAD	−1.578	3.91E-5	−4.151	10	86	Beer lung statistics
	LUAD	−1.709	0.022	−2.155	17	132	Bhattacharjee lung statistics
***ST3GAL3***	NA	NA	NA	NA	NA	NA	NA
***ST3GAL4***	LUAD	2.031	0.018	2.583	6	40	Garber lung statitics Ouyang et al. (2020)
	LUAD	−1.722	0.039	−1.863	17	132	Bhattacharjee lung statistics
***ST3GAL5***	LUAD	1.556	2.33E-5	4.342	58	58	Selamat lung statitics Glavey et al. (2014)
	LUAD	−1.797	0.004	−3.270	6	39	Garber lung statistics
	LUAD	−2.012	0.022	−2.145	17	132	Bhattacharjee lung statistics
***ST3GAL6***	LUAD	−1.914	0.003	−3.254	6	38	Garber lung statistics
	LUAD	−1.955	9.11E-12	−7.785	65	45	Hou lung statistics
	LUAD	−1.618	0.010	−2.418	30	27	Su lung statistics Hu et al. (2019)
	LUAD	−1.860	0.022	−2.140	17	132	Bhattacharjee lung statistics

### Correlation among the expression levels of the ST3GAL family members and tumor stage and molecular subtype in lung adenocarcinoma patients

**FIGURE 2:**
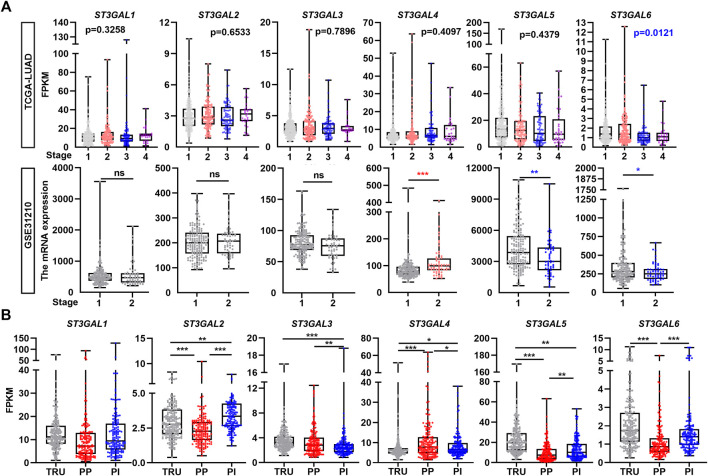
The correlation of the expression levels of the ST3GAL family members with tumor stage and subtype in LUAD samples. **(A)** The expression levels of the ST3GAL family members were correlated with the pathological stages of LUAD patients in the TCGA-LUAD and GSE31210 datasets. Stage 1 (*n* = 278); 2 (*n* = 124); 3 (*n* = 83); and 4 (*n* = 27) of the TCGA-LUAD dataset and stage 1 (*n* = 168) and 2 (*n* = 58) of GSE31210 were indicated. **(B)** The expression levels of the ST3GAL family members were correlated with the molecular subtype of LUAD patients in the TCGA-LUAD dataset. Three molecular subtypes of LUAD were shown as the terminal respiratory unit (TRU; *n* = 207), proximal-proliferative (PP; *n* = 169), proximal-inflammatory (PI; *n* = 159). One-way ANOVA test; unpaired *t* test (two-tailed), ∗, *p* < 0.05; ∗∗, *p* < 0.01; ∗∗∗, *p* < 0.001; ns: no significance.

Next, we chose the TCGA-LUAD and GSE31210 datasets to examine the association among ST3GAL family members and clinicopathological stages. As shown in Figure 2A, the expression level of ST3GAL6 was consistently and negatively associated with the stage of LUAD patients, while there was no consistent association among the expression of the other five family members and pathological stages from the two independent datasets. In the TCGA-LUAD dataset, three molecular subtypes, including the TRU, PP and PI, were identified with different clinical outcomes (Cancer Genome Atlas Research Network, 2014). Hence, by comparing the mRNA levels of the ST3GAL family members in three molecular subtypes, we found that the expression levels of these six genes had statistical differences among the molecular subtypes (Figure 2B). Particularly, the expression levels of ST3GAL2, 5, and 6 were significantly reduced in the PP subtype with poor prognosis, but had high levels in TRU, which shows the best clinical outcome among the three molecular subtypes (Cancer Genome Atlas Research Network, 2014). On the contrary, ST3GAL4 had a significant increase in mRNA levels in the PP subtype and was downregulated in the TRU subtype (Figure 2B).

### Prognostic indicator of the ST3GAL family members in lung adenocarcinoma patients

**FIGURE 3:**
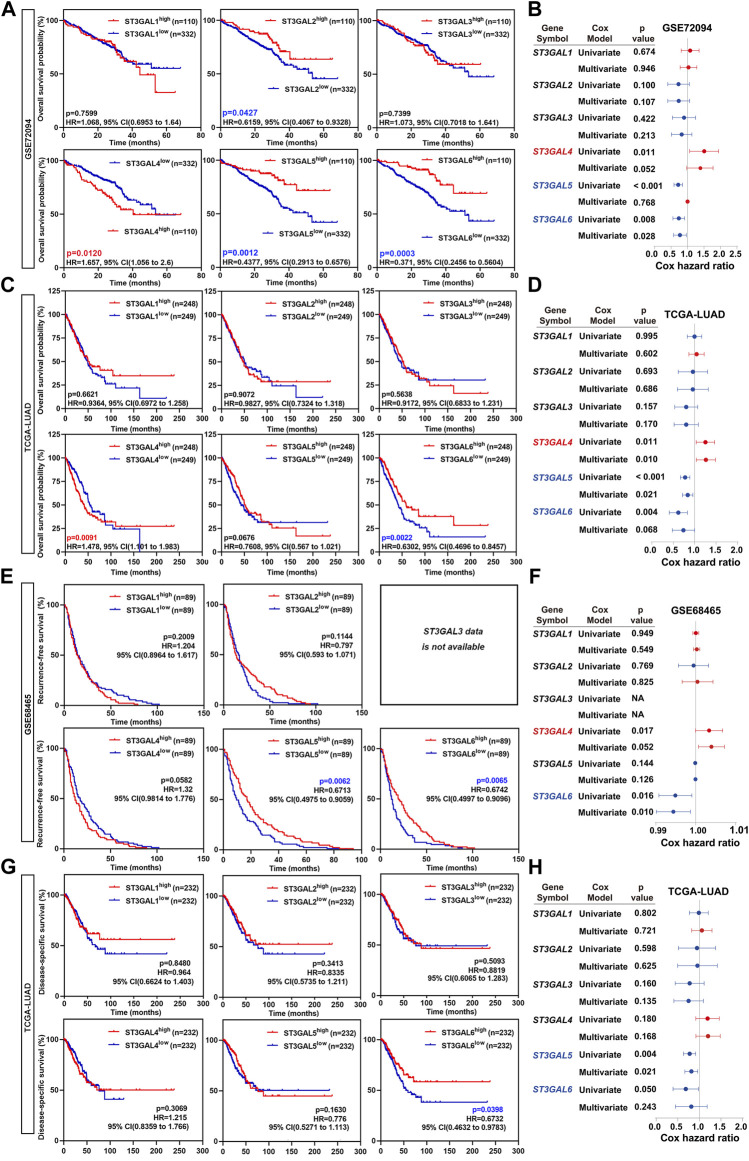
The overall survival rate of the ST3GAL family members in LUAD patients. **(A)** Kaplan–Meier plots analyzing the OS rates of LUAD patients with high- or low-expression levels of the ST3GAL family members from the GSE72094 dataset. **(B)** Forest plots demonstrating the univariate Cox regression and multivariate Cox analyses of the ST3GAL family members based on the overall survival (OS) in the GSE72094 dataset. **(C)** Kaplan–Meier plots analyzing the OS rates of LUAD patients with high- or low-expression levels of the ST3GAL family members from the TCGA-LUAD dataset. **(D)** Forest plots demonstrating the univariate Cox regression and multivariate Cox analyses of the ST3GAL family members, based on the OS in the TCGA-LUAD dataset. **(E)** The Kaplan–Meier plot analyzing recurrence-free survival (RFS) rates in LUAD patients with high- or low-expression levels of the ST3GAL members from the GSE48465 dataset. **(F)** Forest plot demonstrating the univariate Cox regression and multivariate Cox analyses of the ST3GAL members based on RFS in the GSE48465 dataset. **(G)** The Kaplan–Meier plot analyzing disease-specific survival (DSS) rates in LUAD patients with high- or low-expression levels of the ST3GAL members from the TCGA-LUAD dataset. **(H)** Forest plot demonstrating the univariate Cox regression and multivariate Cox analyses of the ST3GAL members, based on DSS in the TCGA-LUAD dataset.

To further assess whether the six members of the ST3GAL family can be used as an independent prognostic factor, Kaplan–Meier, univariate, and multivariate Cox regression analyses were conducted. The Kaplan–Meier analysis revealed that the downregulation of ST3GAL2, 5, and 6, and the upregulation of ST3GAL4 predicted worse clinical outcomes in LUAD patients in the GSE72094 dataset (Figure 3A). As for the OS analysis in the GSE72094 dataset, ST3GAL4 (*p* = 0.011, HR = 1.259, 95% CI = 1.056–1.501), ST3GAL5 (*p* < 0.001, HR = 0.70, 95% CI = 0.593–0.848), and ST3GAL6 (*p* = 0.008, HR = 0.720, 95% CI = 0.564–0.919) revealed to be associated with the OS in the univariate analysis, while only ST3GAL6 (*p* = 0.028, HR = 0.755, 95% CI = 0.588–0.970) was still significantly related to the OS in the multivariate Cox analysis (Figure 3B). In an independent TCGA-LUAD dataset, the Kaplan–Meier analysis revealed that the downregulation of ST3GAL6 and the upregulation of ST3GAL4 predicted worse clinical outcomes in LUAD patients in the TCGA-LUAD dataset (Figure 3C). At the same time, ST3GAL4 (*p* = 0.011, HR = 1.25, 95% CI = 1.052–1.480), ST3GAL5 (*p* < 0.001, HR = 0.786, 95% CI = 0.684–0.903), and ST3GAL6 (*p* = 0.004, HR = 0.611, 95% CI = 0.436–0.855) had significant relevance to OS in the univariate analysis and only ST3GAL4 (*p* = 0.010, HR = 1.259, 95% CI = 1.056–1.501) and ST3GAL5 (*p* = 0.021, HR = 0.844, 95% CI = 0.731–0.975) were significantly related to the OS (*p* < 0.05) in the multivariate Cox analysis (Figure 3D).

For further investigating the prognostic critical efficiency of the ST3GAL family members in LUAD patients, we performed the multivariate Cox regression analyses and the Kaplan–Meier survival curve with the log-rank test according to RFS and DSS based on the GSE68465 and TCGA-LUAD datasets. As for the RFS analysis, the Kaplan–Meier analyses revealed that low ST3GAL5 and 6 levels predicted a significantly shorter RFS time in LUAD patients (GSE68465 dataset; Figure 3E). Two genes were related to RFS in the univariate analysis: ST3GAL4 (*p* = 0.017, HR = 1.004, 95% CI = 1.001–1.007) and ST3GAL6 (*p* = 0.016, HR = 0.995, 95% CI = 0.991–0.999), but only ST3GAL6 (*p* = 0.010, HR = 0.994, 95% CI = 0.990–0.999) was proved to be significantly related to RFS (*p* < 0.05) in the multivariate Cox analysis (Figure 3F). As for the DSS analysis, a low expression of ST3GAL6 predicts a shorter DSS time in LUAD patients (TCGA-LUAD dataset; Figure 3G). Similarly, ST3GAL5 (*p* = 0.004, HR = 0.767, 95% CI = 0.639–0.920) and ST3GAL6 (*p* = 0.050, HR = 0.645, 95% CI = 0.415–1.000) revealed to be associated with DSS in the univariate analysis and only ST3GAL5 (*p* = 0.021, HR = 0.806, 95% CI = 0.672–0.968) was significantly related to OS in the multivariate Cox analysis (Figure 3H).

In summary, we concluded that ST3GAL4, ST3GAL5, and ST3GAL6 may be potential factors predicting the prognosis in LUAD patients, among which, ST3GAL5 and ST3GAL6 could be used as indicators of a favorable prognosis, and ST3GAL4 may be used as an indicator of a poor prognosis. Taken together, ST3GAL6 has the most robust prognostic performance among the ST3GAL family members.

### Genetic alteration analysis of six ST3GAL members and a PPI analysis on ST3GAL6 in lung adenocarcinoma patients

**FIGURE 4:**
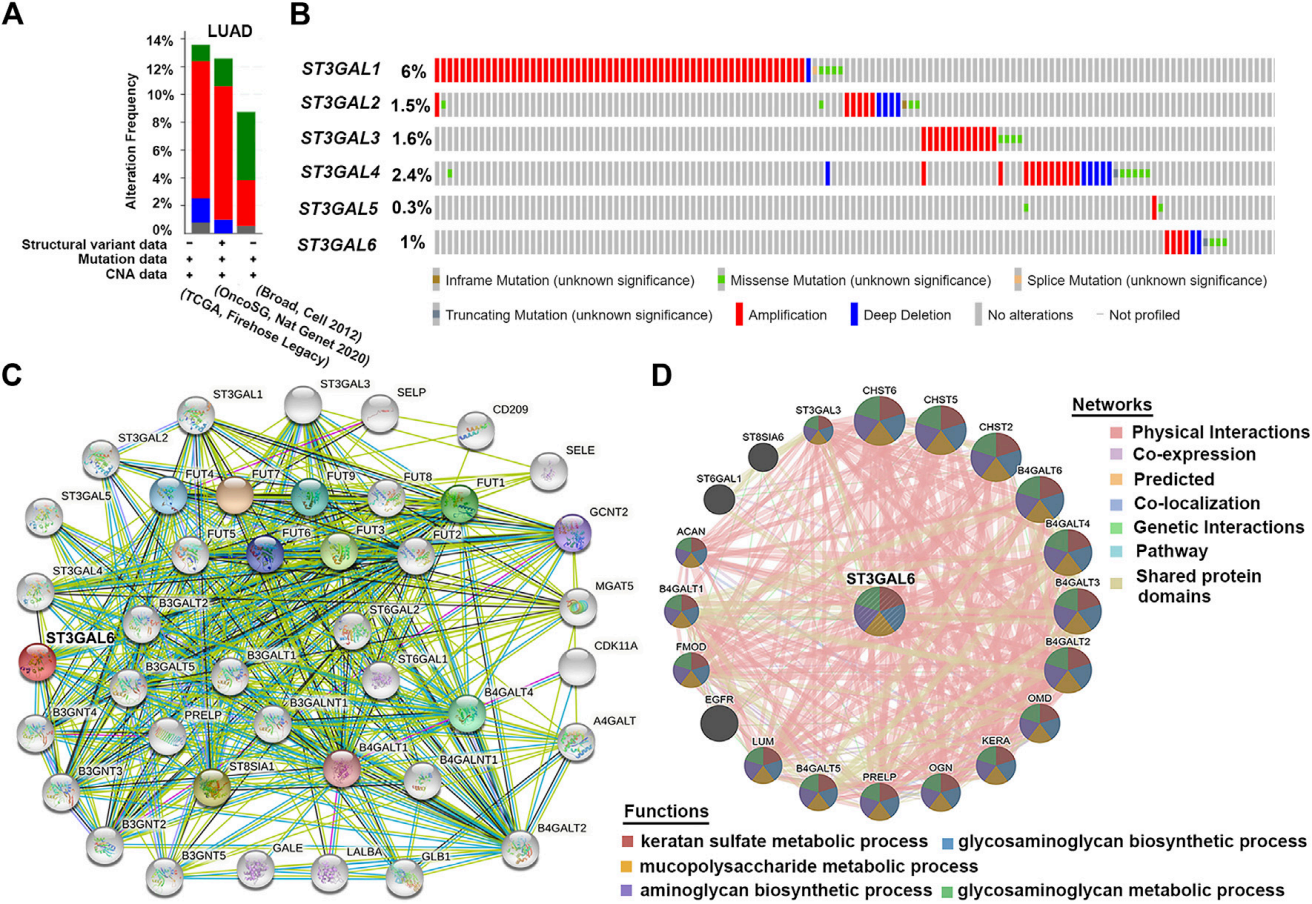
The genetic alterations of the ST3GAL family members with PPI analyses of ST3GAL6 in LUAD samples. **(A,B)** Genetic alterations of the ST3GAL family members in LUAD were analyzed in plots **(A)** and in individual cases **(B)** by the cBioPortal online database. **(C,D)** PPI network analysis of ST3GAL6 was performed through STRING **(C)** and GeneMANIA **(D)**.

The cBioPortal online tool for LUAD patients was used to analyze the genetic alteration frequency and the types of the ST3GAL family members. As shown in Figure 4A, we analyzed the overall mutation frequency and copy number changes of ST3GALs in three LUAD datasets including the Broad dataset (Imielinski et al., 2012), OncoSG dataset (Chen et al., 2020), and TCGA-LUAD (Firehose Legacy). The result indicated that the overall genetic alteration frequency of the ST3GAL family was not significant with only 13.57% in TCGA-LUAD. Among them, the genetic alteration type with the largest proportion was amplification (51 of 516 cases, 9.88%), the second was deep deletion (9 cases, 1.74%), the least was mutation (6 cases, 1.16%), and the remaining were multiple alterations (4 cases, 0.78%). The specific alteration information of each gene in the three datasets demonstrated that ST3GAL1 showed the highest alteration frequency (6%) among the six family members in LUAD patients (Figure 4B). ST3GAL6 was ranked fifth with 1% of LUAD patients with genetic alterations. The remaining four genes, ST3GAL2, ST3GAL3, ST3GAL4, and ST3GAL5, also had low mutation frequencies, and their mutation rates were 1.5%, 1.6%, 2.4%, and 0.3%, respectively.

Due to the low mutation rate of the ST3GAL family in general, we conducted the analysis from the perspective of protein–protein interaction. Based on the previous results, we used the STRING database to perform the PPI network interaction analysis centering on ST3GAL6 in the family that we were interested in. The results showed that most proteins associated with ST3GAL6 were glyco-metabolism-related proteins, such as FUT family members and B4GALTs, etc. (Figure 4C). Next, the results of the GeneMANIA database showed that ST3GAL6 was involved in keratan sulfate metabolic, glycosaminoglycan biosynthetic, and mucopolysaccharide metabolic processes. In addition to the known glycometabolism-related pathways, we also found that ST3GAL6 was associated with the EGFR signaling pathway, suggesting that ST3GAL6 may be involved in EGFR signaling in LUAD cells (Figure 4D). In summary, these results showed that the ST3GALs had low genetic alteration rates and the potential association of EGFR and ST3GAL6.

### Function enrichment, pathway analysis of ST3GAL6 in lung adenocarcinoma patients

Since the aforementioned data consistently demonstrated that ST3GAL6 had the best clinical and molecular relevance in LUAD patients among the ST3GAL family members across several public datasets, we next tried to investigate the functions and pathways of ST3GAL6 in LUAD. First, by utilizing the significant ST3GAL6-associated genes in the TCGA-LUAD dataset, we performed GO enrichment and KEGG analyses. Among the 30 most highly enriched functions in the GO items, ST3GAL6-associated genes were involved in the regulation of the immune system, such as antigen processing and presentation, neutrophil activation involved in immune response, as well as mitotic nuclear division in the biological processes (BP) category. In the cellular components (CC) part, the genes were mainly enriched in the MHC class II protein complex, mitotic spindle, condensed chromosome, centromeric region, and external side of the plasma membrane, etc. The mostly affected molecular function (MF) included the MHC class II receptor activity, carbohydrate binding, and Rho GTPase binding, etc. (Supplementary Figure S1A).

Furthermore, the KEGG pathway analysis of ST3GAL6 in LUAD demonstrated that 19 pathways were significantly related to ST3GAL6 and co-expressed genes, including an intestinal immune network for IgA production, cell adhesion molecules, NOD-like receptor signaling pathway, antigen processing and presentation, and inflammatory bowel disease, as well as cell adhesion molecules (Supplementary Figure S1B). These results suggest that ST3GAL6 may also have other biological functions during the development of LUAD.

### ST3GAL6 predicts a better prognosis in lung adenocarcinoma patients

**FIGURE 5:**
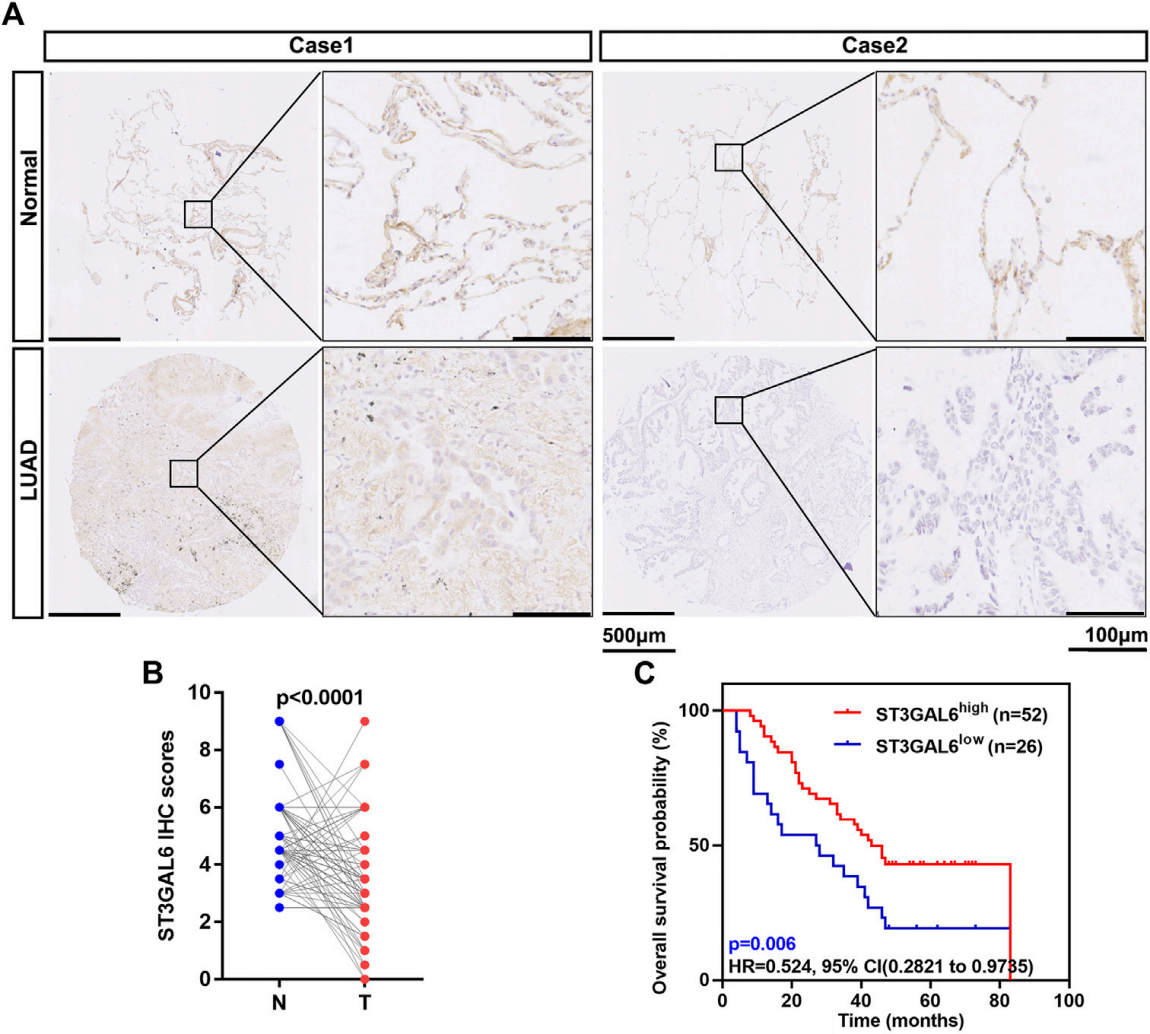
Low expression of ST3GAL6 in LUAD patients is associated with a poorer prognosis. **(A)** The representative IHC images of the ST3GAL6 expression in LUAD and normal tissues. **(B)** The ST3GAL6 expression levels in 88 paired LUAD tissues (T) and their adjacent normal tissues (N) through their IHC score. **(C)** The Kaplan–Meier plot analyzing the OS rates of LUAD patients from the IHC cohort with a IHC score (*n* = 78). Scale bar, 500 and 100 μm. Paired *t* test (two-tailed).

In our previous public database analysis, a low ST3GAL6 mRNA level in LUAD was found to predict a poor prognosis. To further validate this result, we performed IHC staining for ST3GAL6 in a commercial tissue microarray containing 88 paired LUAD and adjacent normal lung specimens, with 4 unpaired LUAD specimens. The ST3GAL6 protein was strongly detected in the cytosol of lung epithelial cells in two normal tissues from two LUAD patients (Figure 5A). In the LUAD specimens, ST3GAL6 was slightly reduced in lung cancer cells from a LUAD patient with stage 1 (case 1), while ST3GAL6 was barely detected in the lung cancer cells from a LUAD patient with stage 3 (case 2). Consistent with the previous results from public databases, the ST3GAL6 protein level was significantly lower in LUAD tissues than in the paired normal lung tissues (*n* = 88; Figure 5B). Of note, ST3GAL6 predicted a better prognosis in LUAD patients (Figure 5C). Overall, these data reinforced the notion that ST3GAL6 was downregulated in LUAD samples compared with normal lung tissues, and LUAD patients with low ST3GAL6 had a short survival time.

### ST3GAL6 knockdown leads to the activation of the ERK pathway and cell invasion in LUAD cells

**FIGURE 6:**
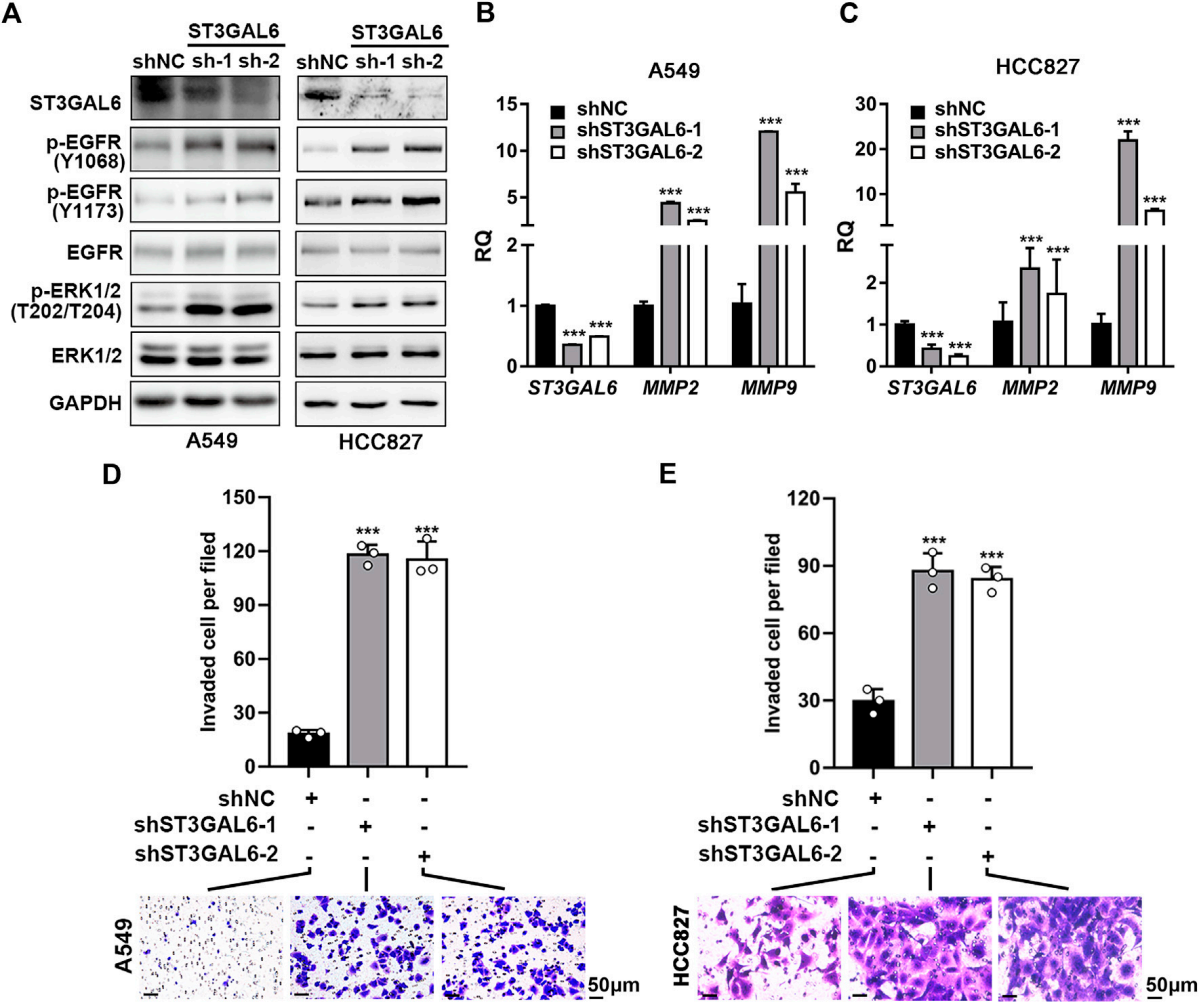
Knocking down ST3GAL6 activates EGFR signaling and promotes the cancer cell invasion capacities of A549 and HCC827 cells. **(A)** Western blotting to investigate the activation of EGFR signaling in ST3GAL6 knockdown cancer cells. **(B,C)** The mRNA expression levels of ST3GAL6, MMP2, and MMP9 in A549 **(B)** and HCC827 cells **(C)** with ST3GAL6 knockdown. **(D,E)** Transwell assay on the cell invasion capacities of A549 **(D)** and HCC827 cells **(E)** with ST3GAL6 knockdown. Scale bar, 50 μm. Data are presented as mean ± SD of three independent experiments; Unpaired *t* test (two-tailed),∗∗∗*p* < 0.001.

**FIGURE 7:**
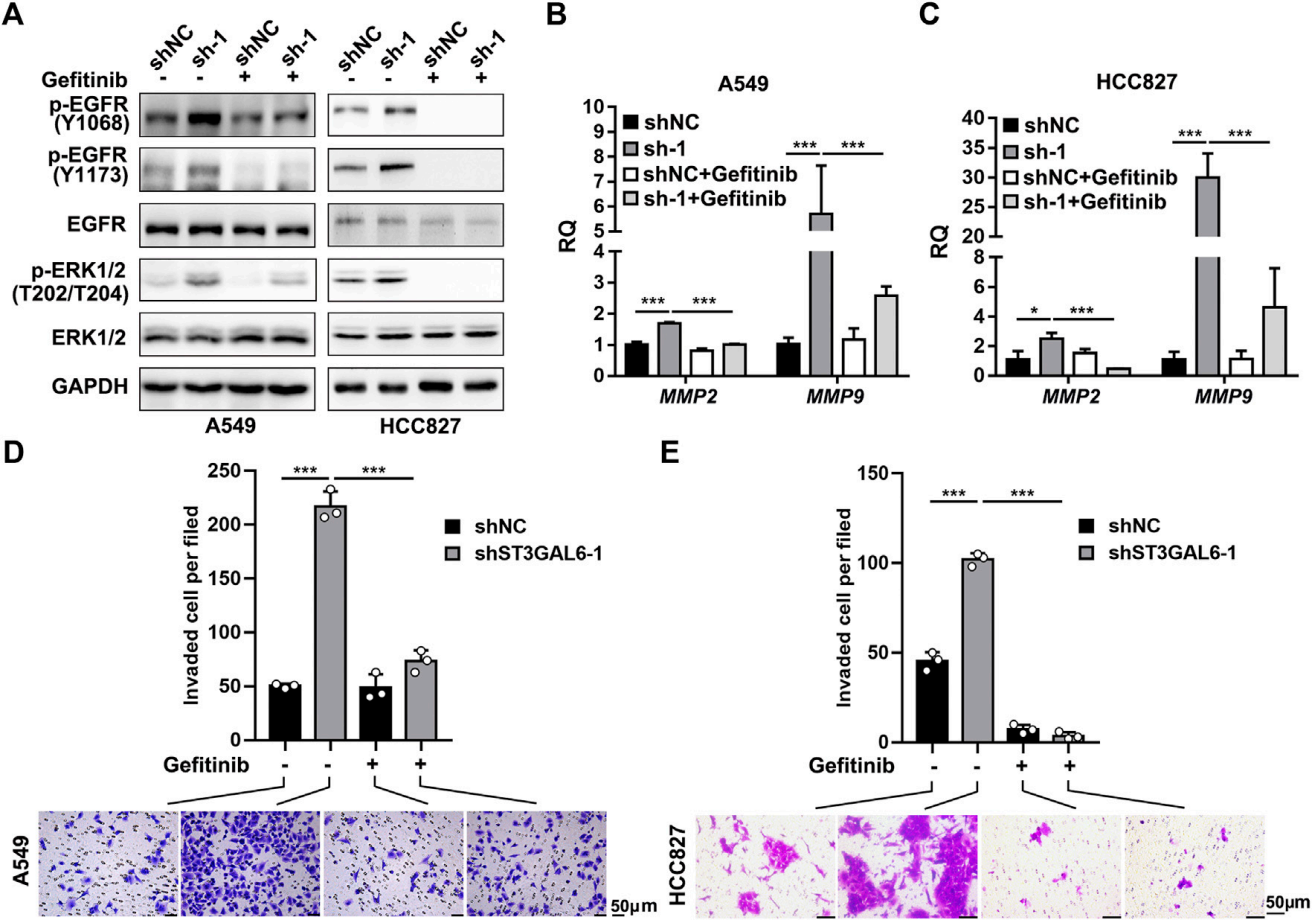
Gefitinib inhibits the EGFR signaling pathway activation and cell invasion induced by ST3GAL6 deletion in A549 and HCC827 cell lines. **(A)** Western blotting to investigate the activation of EGFR signaling in ST3GAL6 knockdown cancer cells treated with Gefitinib. A549 and HCC827 cells were treated with 5 and 1 µM of Gefitinib for 24 h, respectively **(B,C)** The mRNA expression levels of MMP2 and MMP9 in A549 **(B)** and HCC827 **(C)** ST3GAL6 knockdown cells treated with Gefitinib. A549 and HCC827 cells were treated with 5 and 1 µM of Gefitinib for 48 and 24 h **(D,E)** Transwell assay on the cell invasion capacities of A549 **(D)** and HCC827 **(E)** ST3GAL6 knockdown cells treated with Gefitinib. A549 and HCC827 cells were treated with 5 and 1 µM of Gefitinib for 24 and 16 h, respectively. Scale bar, 50 μm. Data are presented as mean ± SD of three independent experiments; Unpaired *t* test (two-tailed), ∗, *p* < 0.05; ∗∗∗*p* < 0.001.

To explore the biological function of ST3GAL6 in the LUAD development, we first selected LUAD cell line A549 with wild-type EGFR expression and an EGFR-mutant (exon 19 deletion) LUAD cell line HCC827 (Tracy et al., 2004) to deplete the endogenous ST3GAL6 expression, according to the expression level of ST3GAL6 in a non-small cell lung cancer cell line from the CCLE database for STG3AL6 knockdown by two short hairpin RNAs (shRNAs) (Supplementary Figure S2A). Since sialylation of EGFR is reported to counteract with the phosphorylation of EGFR (Liu et al., 2011), we examined the EGFR activation by its phosphorylation levels, as well as its downstream direct targets. As shown in Figures 6A–C, compared with control cells (shNC), the phosphorylation levels of EGFR at Y1068 and Y1173, which are related to the activation of EGFR signaling (Gao et al., 2005), were both strikingly elevated in stable ST3GAL6 knockdown A549 and HCC827 cells. Previous studies have shown that ST3GAL6 is involved in the regulation of the PI3K/AKT signaling pathway in colorectal cancer cells (Hu et al., 2019), but our results showed that ST3GAL6 knockdown did not affect this pathway in LUAD cells (Supplementary Figures S2B,C). Instead, we observed the increased level of phosphorylated ERK1/2, indicating that the depletion of ST3GAL6 activates the EGFR/MAPK signaling pathway. The matrix metalloproteinases (MMPs) are well-known AP-1 transcription factor target genes, which are activated by the MAPK signaling pathway (Yan et al., 2008; Lee et al., 2013; Shen et al., 2017). Hence, we detected the remarkably elevated expression levels of two well-known target genes of EGFR/MAPK/AP1 signaling, MMP2, and MMP9 in ST3GAL6 knockdown cells. Since the activation of EGFR signaling may be involved in cancer cell invasion, we carried out a transwell assay and found that ST3GAL6 knockdown could significantly increase cancer cell invasiveness (Figures 6D,E). In order to prove that the EGFR signaling pathway is the key signaling pathway that promotes the invasion of LUAD cells after ST3GAL6 deletion, we treated these cells with EGFR inhibitor Gefitinib. The results showed that Gefitinib successfully inhibited the upregulation of the MMP2/9 expression (Figures 7A–C) and cell invasion caused by ST3GAL6 deletion (Figures 7D,E). Interestingly, we found that the knockdown of ST3GAL6 increased the resistance of EGFR-tyrosine kinase inhibitor-sensitive HCC827 to Gefitinib, but not the Gefitinib-insensitve A549 cells. These data indicated that the lower expression of ST3GAL6 might decrease the effect of EGFR inhibitors in LUAD cells with EGFR inhibitor-sensitive mutations (Supplementary Figures S3A–D). Meanwhile, SB-3CT, an inhibitor of MMP2/9, was added to A549 cells with ST3GAL6 depletion, and the results showed that the invasion ability of A549 cells was significantly reduced after SB-3CT treatment (Supplementary Figure S4). The results indicated that cell invasion induced by ST3GAL6 deletion is mainly due to the upregulation of MMP2/9. These results indicated that ST3GAL6 deficiency induced the activation of the EGFR/MAPK signaling, leading to the transcriptional activation of its target genes, *MMP2* and *9*, which may account for the increased capacity of LUAD cell invasiveness.

### ST3GAL6-AS1 regulates the expression of ST3GAL6 in lung adenocarcinoma

**FIGURE 8:**
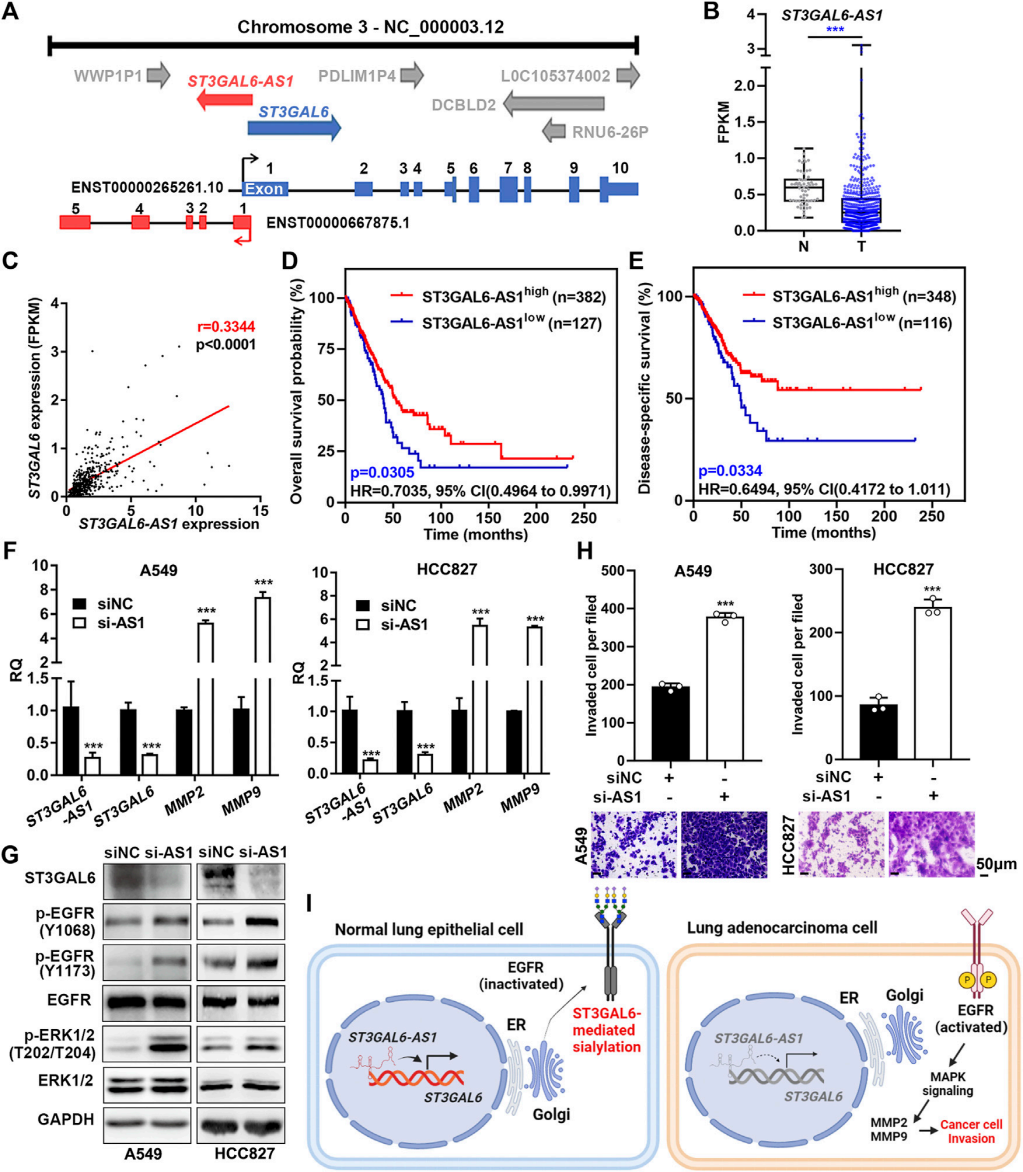
ST3GAL6-AS1 regulates the expression of ST3GAL6 in LUAD cells. **(A)** Schematic diagram of the genomic structure of the ST3GAL6 and ST3GAL6-AS1 genes. **(B)** The expression levels of ST3GAL6-AS1 in tumor (T) and non-tumor (N) in the TCGA-LUAD datasets. T, Tumor tissues; N, Normal tissues; TCGA-Normal (*n* = 59), TCGA-Tumor (*n* = 535). **(C)** Correlation between ST3GAL6 and ST3GAL6-AS1 expressions from the TCGA-LUAD dataset by the Pearson correlation analysis. **(D,E)** The Kaplan–Meier plot analyzing the overall survival **(D)** and disease-specific survival **(E)** in patients with high- or low-expression levels of ST3GAL6-AS1 from the TCGA-LUAD datasets. **(F)** mRNA expression levels of ST3GAL6-AS1, ST3GAL6, MMP2, and MMP9 in A549 and HCC827 cells with the ST3GAL6-AS1 knockdown. **(G)** Western blotting assays on the changes of EGFR/ERK signaling in ST3GAL6-AS1-depleted A549 and HCC827 cells. **(H)** Transwell assay on the invasion capacities of A549 and HCC827 cells with ST3GAL6-AS1 knockdown. Scale bar, 50 μm. **(I)** The working model of ST3GAL6 in LUAD cells. Data were presented as mean ± SD of the three independent experiments; Unpaired *t* test (two-tailed), ∗∗∗*p* < 0.001.

To further dissect the downregulation of ST3GAL6 in LUAD samples, we found that there exists a long non-coding RNA (lncRNA), ST3GAL6-AS1, overlapping with the first exon of the ST3GAL6 gene (Figure 8A). Since antisense lncRNAs have been reported to regulate their host gene expression (Magistri et al., 2012; Zhuang et al., 2015), with currently no reports on the role of ST3GAL6-AS1 in LUAD, we first carried out a bioinformatics analysis and found that ST3GAL6-AS1 was downregulated in LUAD samples, compared with normal lung tissues in the TCGA-LUAD dataset (Figure 8B). Interestingly, the expression levels between ST3GAL6 and ST3GAL6-AS1 were positively correlated in LUAD samples, suggesting the potential regulation between this lncRNA and its host gene (Figure 8C). Of note, the low expression of ST3GAL6-AS1 was associated with the poor prognosis and short DSS duration of the disease (Figures 8D,E). Next, we successfully depleted ST3GAL6-AS1 in A549 and HCC827 cells and observed the downregulation of ST3GAL6 at the mRNA and protein levels (Figures 8F,G). Similar to the observation in ST3GAL6 knockdown cells, we found elevated phosphorylation levels of EGFR at Y1068 and Y1173, and downstream ERK at T202/T204 in ST3GAL6-AS1-depleted A549 and HCC827 cells (Figure 8G), accompanied by the significantly increased mRNA levels of MMP2 and MMP9. As expected, the knockdown of ST3GAL6-AS1 significantly promoted the invasion capabilities of A549 and HCC827 cells (Figure 8H). In summary, we found that ST3GAL6-AS1 positively regulates ST3GAL6 in LUAD cells, while the depletion of ST3GAL6-AS1 activates the EGFR signaling pathway, upregulates the expressions of MMP2 and 9, and promotes the invasion of LUAD tumor cells.

## Discussion

Aberrant sialylation has been implicated in the development of many cancers, associated with various cancer hallmarks (Dall’Olio et al., 2014; Schjoldager et al., 2020; Dobie and Skropeta, 2021). Herein, by systematically analyzing the association of ST3GAL family members with various clinicopathological parameters of LUAD samples in multiple independent datasets, we successfully identified that ST3GAL6 is the most potential prognostic biomarker among the six ST3GAL family members. ST3GAL6 is significantly downregulated in the proximal-proliferative (PP) molecular subtype with a worse clinical outcome. Finally, we found that the depletion of ST3GAL6 in LUAD cells induced the phosphorylation of the EGFR and promoted the activation of the downstream MAPK signaling pathway, ultimately leading to enhanced invasion capacities of LUAD cells. At the same time, we found the upstream ST3GAL6-AS1 that regulated the expression of ST3GAL6 (Figure 8I). In addition, we found that the depletion of ST3GAL6-AS1 recapitulated the effects of ST3GAL6 deficiency in LUAD cells.

The ST3GAL family members catalyze the formation of α2,3-linked sialylation onto terminal Gal residues of glycoproteins and glycolipids (Pearce and Läubli, 2016). Hence, the dysregulation of ST3GALs, which has been implicated in various cancer types, accounts for the aberrant sialylation in tumors. As one of the most studied ST3GAL members, ST3GAL1 is overexpressed in glioblastoma, melanoma, and estrogen receptor-positive breast cancer, through regulating cancer stemness, metastasis, angiogenesis, and tumor immune evasion (Chong et al., 2016; Pietrobono et al., 2020; Lin et al., 2021). Several substrates were identified to be modified with sialylation by ST3GAL1, including the membrane receptors (AXL, GFRA1, and CD55), and secreted protein (vasorin) (Fan et al., 2018; Pietrobono et al., 2020; Lin et al., 2021). In addition to ST3GAL1, the elevated levels of ST3GAL2 and its product sialyl-glycolipid stage-specific embryonic antigen 4 (SSEA4) were suggested for the increased chemoresistance of triple-negative breast cancer to genotoxic agents (Aloia et al., 2015). The overexpressed ST3GAL3 and ST3GAL4 in pancreatic cancer increase the biosynthesis of the sialic acid-Lewis (sLe) antigen, thus promoting tumor metastasis (Guerrero et al., 2020). Though ST3GAL5 overexpression is required for breast cancer stemness (Liang et al., 2013), its downregulation leads to muscle invasion and a worse prognosis in bladder cancer patients, suggesting that the role of ST3GAL5 is cellular context-dependent (Ouyang et al., 2020). Similar to the cancer type-dependent role of ST3GAL5, ST3GAL6 is upregulated in liver cancer and multiple myeloma, as well as the basal subtype of bladder cancer (Glavey et al., 2014; Hu et al., 2019; Dalangood et al., 2020), but downregulated in colorectal cancer (Sun et al., 2017). In this study, we found that the downregulation of ST3GAL6 predicts a poor prognosis in LUAD samples, reinforcing the notion that ST3GAL6 may play an oncogenic or tumor-suppressive role which is dependent on the cancer type.

The dysregulation of sialylation by ST3GAL6 affects cell-to-cell interaction and signaling transduction. For example, ST3GAL6 overexpression is required for multiple myeloma cell homing to bone marrow niche and subsequent tumor growth (Glavey et al., 2014), while ST3GAL6 can induce AKT/mTOR signaling to promote hepatocellular carcinoma cell proliferation and invasion (Sun et al., 2017). Interestingly, ST3GAL6 also suppresses the PI3K/AKT signaling pathway in colorectal cancer cells (Hu et al., 2019). In our study, we did not observe the change of PI3K/AKT signaling in ST3GAL6 knockdown LUAD cells. Such a discrepancy in the ST3GAL6-mediated signaling in different cancer cells probably can be elucidated by identification of its key downstream glycoproteins. Herein, based on the negative regulation of EGFR activation by sialylation, we proved that ST3GAL6 is one sialyltransferase responsible for the inhibition on EGFR/MAPK activation in LUAD cells. Hence, it is necessary and intriguing to further identify the potential target glycoproteins of the ST3GAL6 protein in order to fully understand its role in various cancer types.

EGFR signaling is often abnormally activated in LUAD, which is closely related to EGFR mutation and overexpression (da Cunha Santos et al., 2011; Herbst et al., 2018; Planchard, 2020). When EGFR is activated, a variety of transcription factors are activated through the activation of downstream signalings to promote the progression of tumor cells. More recently, post-translational modifications of EGFR have also affected its own phosphorylation. Studies have shown that in lung cancer, the saliac acid sites (N32, N151, and N389) of EGFR are usually close to the surface-binding sites of EGF. Therefore, when EGFR is modified by sialic acid, negatively charged sialic acid will affect its electrostatic interaction with EGF, leading to the inhibition of subsequent signalings (Yen et al., 2015). Previous studies revealed that ST3GAL1 may account for sialylation of EGFR in renal cancer cells, while ST3GAL6 may promote sialylation of EGFR in HeLa cells (Gong et al., 2020; Qi et al., 2020). In this study, we found that ST3GAL1 was not significantly altered in LUAD patients, whereas the knockdown of ST3GAL6 strikingly increased EGFR phosphorylation and promoted the invasion abilities of LUAD cells in both wild-type and mutant EGFR cells. It will be of interest to prove the specific sialylation of the EGFR protein by ST3GAL6 and its direct link with EGFR phosphorylation in future. Overall, our data indicate the frequent downregulation of ST3GAL6 activate the EGFR/MAPK signaling pathway, independent of the EGFR mutation status.

The regulation of ST3GAL6 is regulated by multiple approaches. ST3GAL6 is negatively regulated by miR-26a in liver cancer cells through binding to its 3′UTR region (Sun et al., 2017). Our previous study also revealed that ST3GAL6 can be repressed by a luminal-specific transcription factor, GATA3, in bladder cancer cells (Dalangood et al., 2020). lncRNA ST3GAL6-AS1 is an antisense lncRNA to its host gene ST3GAL6. Herein, we found that their expression levels are associated in LUAD samples, and proved that ST3GAL6-AS1 mediated the positive regulation of ST3GAL6 in LUAD cells. In colorectal cancer cells, ST3GAL6-AS1 can recruit histone methyltransferase MLL1 to the promoter region of ST3GAL6 for histone H3K4me3 modification, thus activating the transcription of ST3GAL6 (Hu et al., 2019), while the interaction of ST3GAL6-AS1 with heterogeneous nuclear ribonucleoprotein A2B1 can also stabilize ST3GAL6 mRNA in multiple myeloma cells (Shen et al., 2021). In the future, it will be interesting to examine how ST3GAL6-AS1 regulates ST3GAL6 in LUAD cells. Altogether, the tight positive regulation of lncRNA-ST3GAL6 on its host gene is elucidated in various cancer types, even if the function of ST3GAL6 in cancer cells is opposite.

## Data Availability

The datasets presented in this study can be found in online repositories. The names of the repository/repositories and accession number(s) can be found in the article/Supplementary Material.
