# Supplementary material for: Comprehensive landscape of the ST3GAL family reveals the significance of ST3GAL6-AS1/ST3GAL6 axis on EGFR signaling in lung adenocarcinoma cell invasion
Source: Front Cell Dev Biol. 2022 Aug 26;10:931132. doi: 10.3389/fcell.2022.931132 (PMC9462654; doi:10.3389/fcell.2022.931132)
Supplement: Supplementary file 1 [file DataSheet1.docx]

Supplementary Material

Comprehensive landscape of ST3GAL family reveals the significance of ST3GAL6-AS1/ST3GAL6 axis on EGFR signaling in lung adenocarcinoma cell invasion

Jiaxuan Li ^1, †‡,^ Yiming Long ^2,3, ‡^, Jingya Sun ^2^, Jiajun Wu ^4^, Xiao He ^2,5^, Simei Wang ^2,3^, Xiongbiao Wang ^1,6^, Xiayi Miao ^1*^, Ruimin Huang ^2,3,5*^and Jun Yan ^1,4*^

^1^ Department of Respiratory Medicine, Putuo Hospital, Shanghai University of Traditional Chinese Medicine, Shanghai, China

^2^ Shanghai Institute of Materia Medica, Chinese Academy of Sciences, Shanghai, China

^3^ University of Chinese Academy of Sciences, Beijing, China

^4^ Department of Laboratory Animal Science, Fudan University, Shanghai, China

^5^ School of Chinese Materia Medica, Nanjing University of Chinese Medicine, Nanjing , China

^6^ Interventional Cancer Institute of Chinese Integrative Medicine, Shanghai University of Traditional Chinese Medicine, Shanghai, China

**^‡^** These authors contributed equally to this work.

**^†^** Current affiliation: Shanghai Institute of Infectious Disease and Biosecurity, School of Public Health, Fudan University. Shanghai, China.

*** Correspondence:**Ruimin Huang; Xiayi Miao; Jun Yan
[rmhuang@simm.ac.cn](mailto:rmhuang@simm.ac.cn); [miaoxiayi@shutcm.edu.cn](mailto:miaoxiayi@shutcm.edu.cn); [yan_jun@fudan.edu.cn](mailto:yan_jun@fudan.edu.cn)

# 1.Supplementary Table

**SUPPLEMENTARY** **TABLE 1** | List of oligonucleotide sequences and reagents.

| **Item** | **Direction** | **Sequences (5'-3')** |  |  |
| --- | --- | --- | --- | --- |
| **qRT-PCR primers** |  |  |  |  |
| human ST3GAL6 | Forward | ACTGCATTGCATATTATGGGGAA |  |  |
|  | Reverse | TGGCTTTGATAAACAAGGCTGG |  |  |
| human ST3GAL6-AS1 | Forward | AAGGACCTGAGGATGAAGCC |  |  |
|  | Reverse | GGATTCTGTGCTGCCAGTTG |  |  |
| human MMP2 | Forward | TACAGGATCATTGGCTACACACC |  |  |
|  | Reverse | GGTCACATCGCTCCAGACT |  |  |
| human MMP9 | Forward | GGGACGCAGACATCGTCATC |  |  |
|  | Reverse | TCGTCATCGTCGAAATGGGC |  |  |
| human GAPDH | Forward | GGAGCGAGATCCCTCCAAAAT |  |  |
|  | Reverse | GGCTGTTGTCATACTTCTCATGG |  |  |
| **siRNA sequences (5'-3')** | | |  |  |
| siST3GAL6-AS1 | CUUCCCAUCUCAAGAUUCUTT | |  |  |
| siNC | UUCUCCGAACGUGUCACGUTT | |  |  |
| **shRNA target sequences (5'-3')** | | |  |  |
| shST3GAL6-1 | TCCTCTATTATGTACTGCATT | |  |  |
| shST3GAL6-2 | GATGAGAACATCAGCGGAATA | |  |  |
| **Antibody** | **Company** | **Cat #** | **RRID** | **Application (Dilution)** |
| AKT | CST | #4691 | AB_915783 | WB (1:1,000) |
| Phospho-Akt (Ser473) | CST | #4060 | AB_2315049 | WB (1:1,000) |
| EGF Receptor | Proteintech | 66455-1-Ig | AB_2881824 | WB (1:1,000) |
| phospho-EGF Receptor (Tyr1068) | CST | #3777 | AB_2096270 | WB (1:1,000) |
| phospho-EGF Receptor (Tyr1173) | CST | #4407 | AB_331795 | WB (1:1,000) |
| **Antibody** | **Company** | **Cat #** | **RRID** | **Application (Dilution)** |
| p44/42 MAPK (ERK1/2) | CST | #4695 | AB_390779 | WB (1:1,000) |
| phospho-p44/42 MAPK  (ERK1/2; Thr202/Tyr204) | CST | #4370 | AB_2315112 | WB (1:1,000) |
| p70 S6 Kinase | CST | #9202 | AB_331676 | WB (1:1,000) |
| Phospho-p70 S6 Kinase (Thr389) | CST | #9234 | AB_2269803 | WB (1:1,000) |
| ST3GAL6 | Proteintech | 13154-1-AP | AB_10646441 | WB (1:1,000) |
| ST3GAL6 | Abcam | ab106527 | AB_10865191 | IHC (1:200) |
| GAPDH | Santa Cruz | SC-47724 | AB_627678 | WB (1:10,000) |
| **Kit and Reagents** | **Company** | | **Cat #** | |
| 24-well Transwell chambers | Guangzhou Jet Bio-Filtration Co., Ltd. | | TCS003024 | |
| BCA kit | Tiangen Biotech (Beijing) Co.m Ltd. | | PA115-02 | |
| ChamQ SYBR qPCR Master Mix | Vazyme | | Q341-02 | |
| Gefitinib | Selleck Chemicals LLC | | S1025 | |
| High-sig ECL Western blotting substrate | Tanon | | 180-501 | |
| HiScript® II Q Select RT SuperMix | Vazyme | | R233-01 | |
| Lipofectamine RNAi MAX Reagent | Thermo Fisher Scientific | | 13778150 | |
| Matrigel® Growth Factor Reduced (GFR) Basement Membrane Matrix, LDEV-free | Corning | | #356230 | |
| MTT | Beyotime | | ST316 | |
| SB-3CT | MedChemExpress | | S1326 | |
| Sulforhodamine B sodium salt | Sigma-Aldrich | | S1402 | |
| Tissue microarray_LUAD specimens | Shanghai Outdo Biotech Company | | HLugA180Su04 | |
| Trizol reagent | Invitrogen | | #15596026 | |

# 2.Supplementary Figure

**
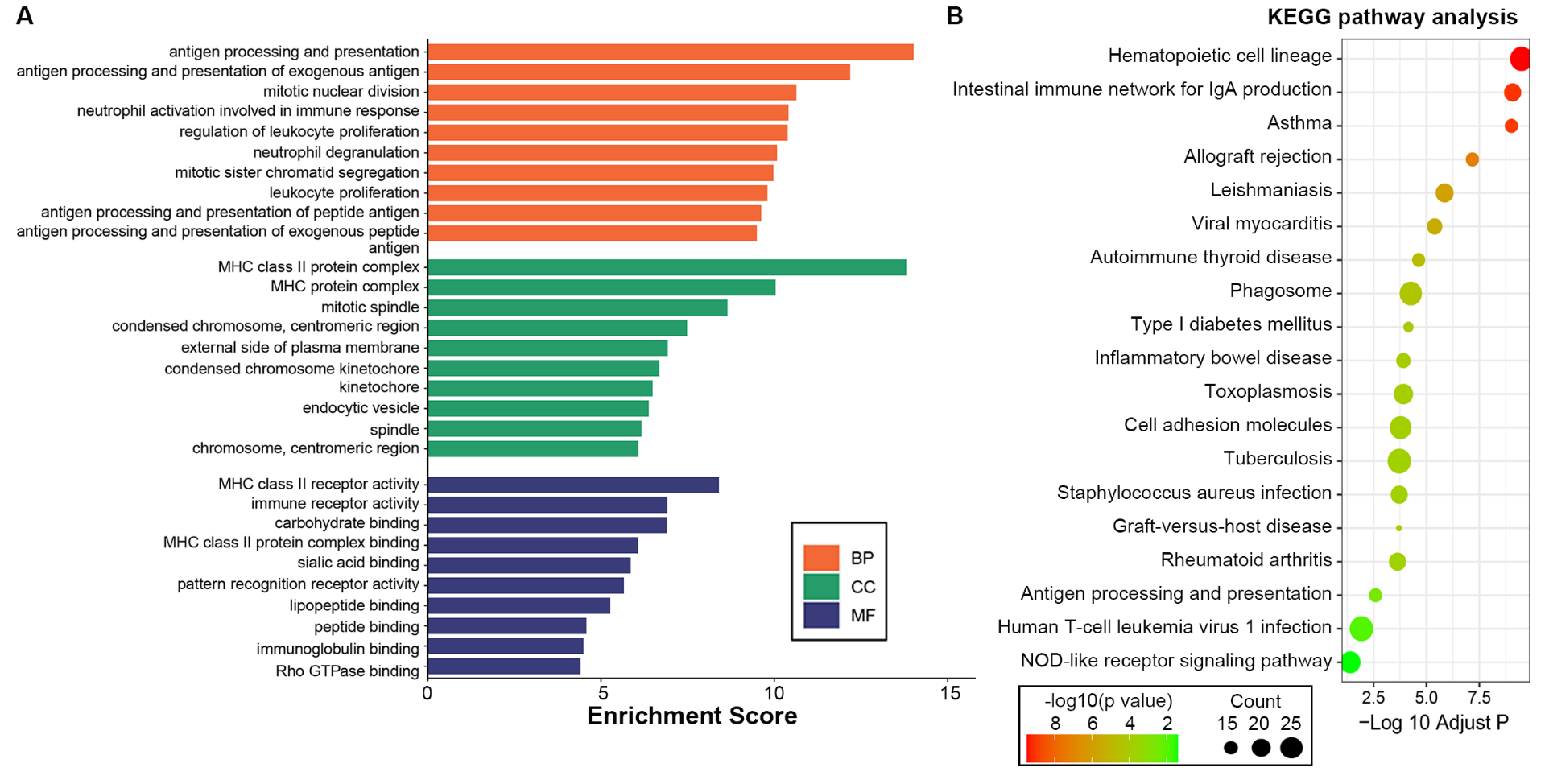
**

**SUPPLEMENTARY** **FIGURE 1** | **GO and KEGG analyses of ST3GAL6 in LUAD samples. (A)** Gene Ontology (GO) enrichment analysis of ST3GAL6 co-expressed genes based on three aspects: biological processes (BP), cellular components (CC) and molecular function (MF). **(B)** Kyoto Encyclopedia of Genes and Genomes (KEGG) analysis of ST3GAL6 co-expressed genes showing pathways significantly associated with ST3GAL6


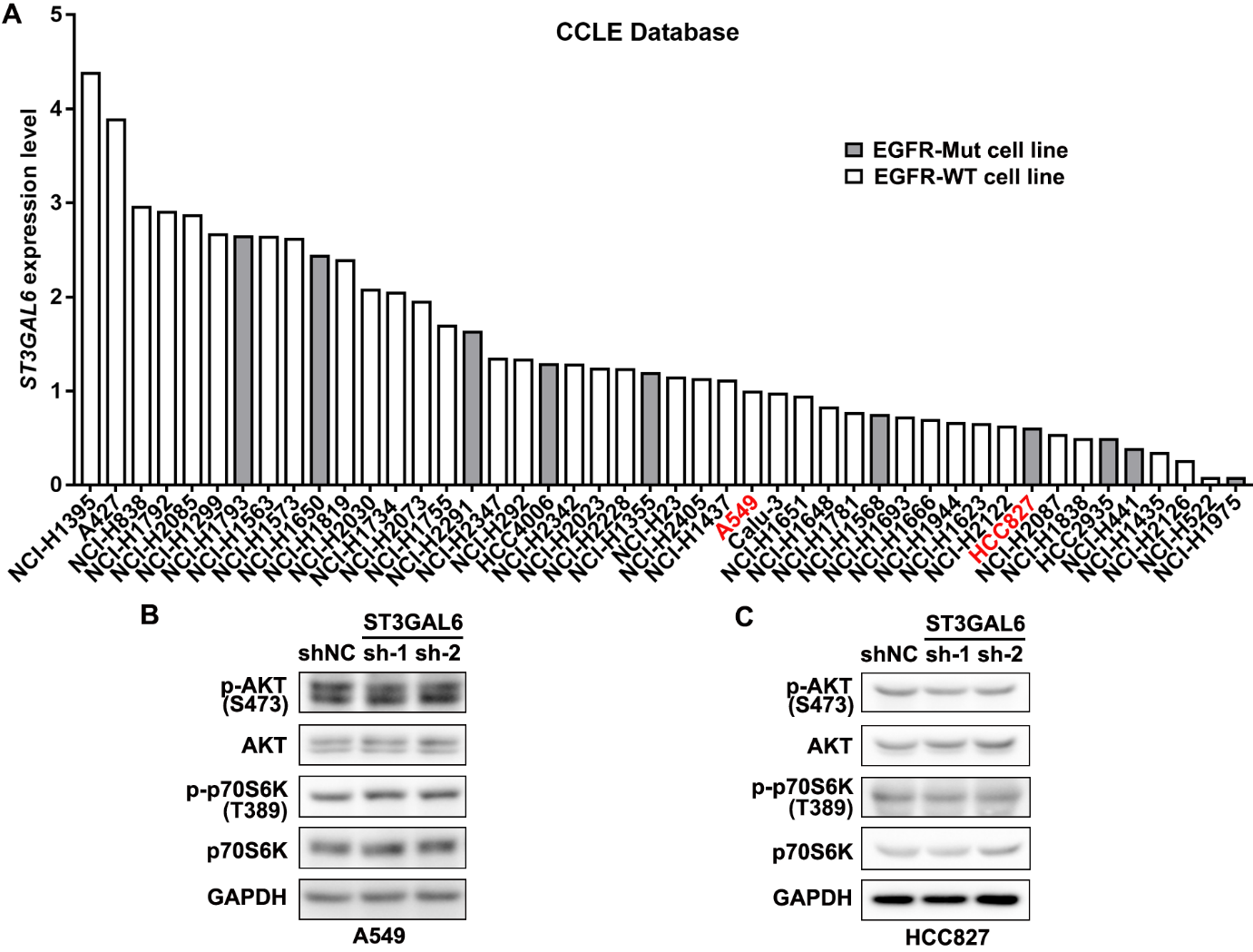


**SUPPLEMENTARY FIGURE 2** | **The expression level of ST3GAL6 in lung cancer cell lines and its effect on PI3K/AKT signaling pathway. (A)** The ST3GAL6 mRNA levels in a panel of non-small cell lung cancer cell lines from Cancer Cell Line Encyclopedia (CCLE) database (<https://sites.broadinstitute.org/ccle/>). A549 and HCC827, which were used in this study, were labelled in red. EGFR-wild-type (WT) lung cancer cell line were depicted as white columns, while EGFR-mutant (Mut) cancer cells were depicted as grey columns. **(B,C)** Western blotting data showing no activation of PI3K/mTOR signaling in ST3GAL6 knockdown (sh-1 and sh-2) A549 **(B)** and HCC827 **(C)** cells, compared to their control cells (shNC).


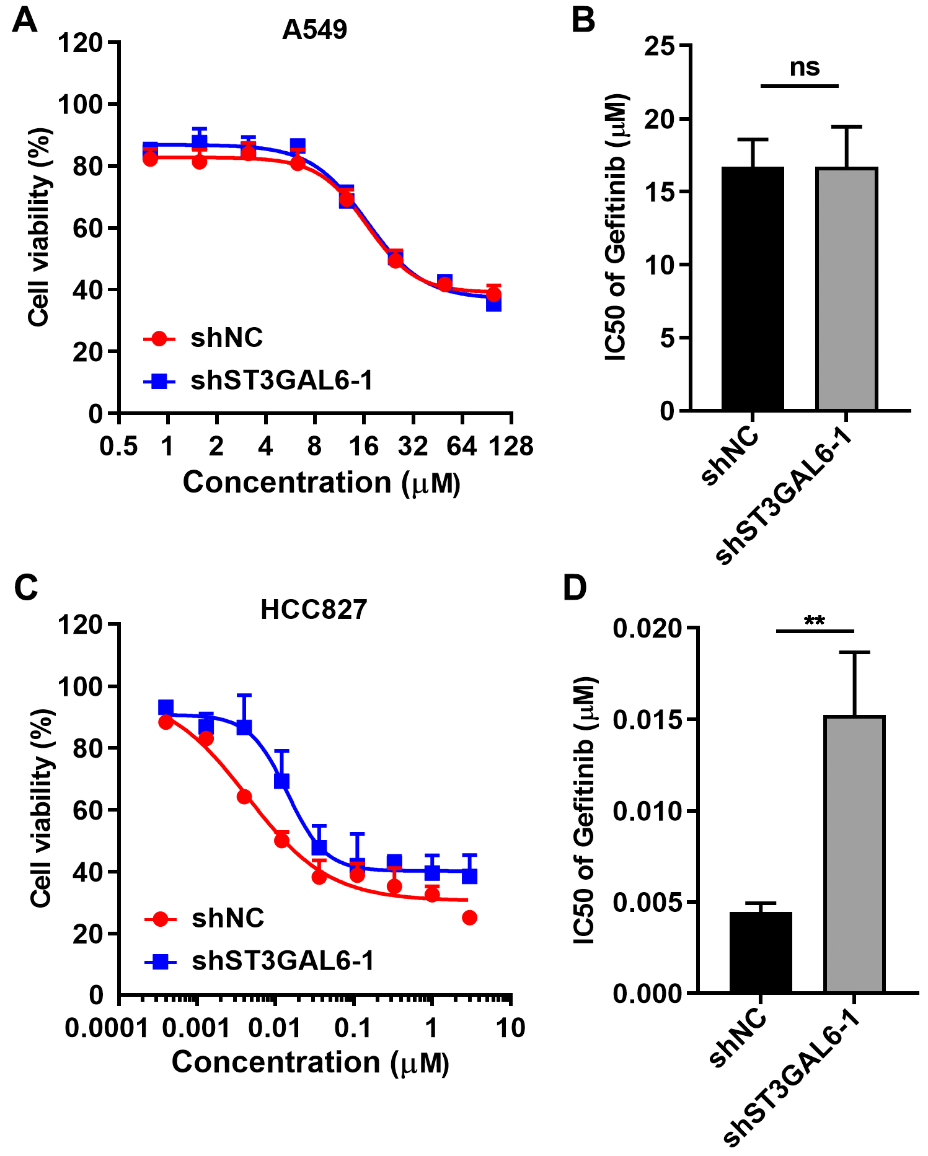


**SUPPLEMENTARY FIGURE 3** | **Cell viability of ST3GAL6 knockdown LUAD cells with gefitinib treatment. (A)** Survival of ST3GAL6 knockdown (shST3GAL6-1) and control (shNC) A549 cells treated with different concentrations of gefitinib for 72 h. **(B)** IC50 values of gefitinib in A549, calculated using GraphPad Prism 7.0. **(C)** Survival of ST3GAL6 knockdown (shST3GAL6-1) and control (shNC) HCC827 cells treated with different concentrations of gefitinib for 72 h. **(D)** IC50 values of gefitinib in HCC827 cells, calculated using GraphPad Prism 7.0. Data were presented as mean ± SD of three independent experiments; Unpaired *t* test (two-tailed), **, p<0.01.

**
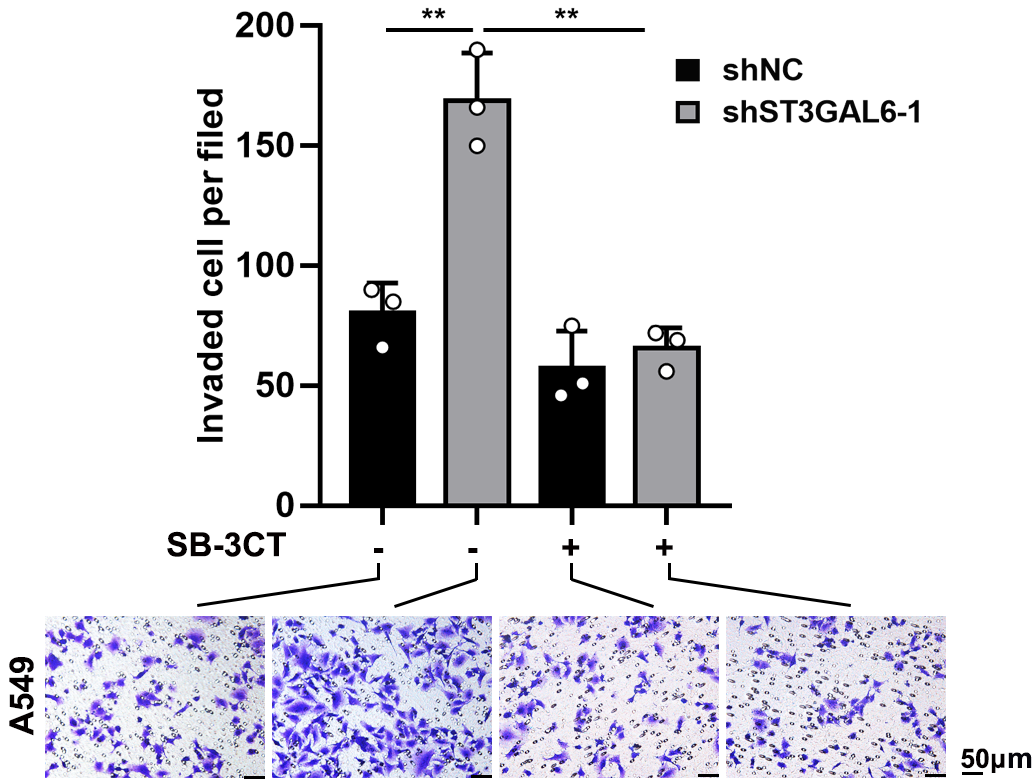
**

**SUPPLEMENTARY FIGURE 4** | **Cell invasion ability of ST3GAL6 knockdown A549 cells with SB-3CT treatment.** Transwell assay on the cell invasion capacities of ST3GAL6 knockdown (shST3GAL6-1) and control (shNC) A549 cells, which were pre-treated with 10 µM SB-3CT and vehicle for 24 h, followed by another 16 h Transwell culture with 10 µM SB-3CT and vehicle, respectively. Scale bar, 50 μm. Data were presented as mean ± SD of three independent experiments; Unpaired *t* test (two-tailed), **, p<0.01.
